# Costs of primary healthcare presentations and hospital admissions for scabies and related skin infections in Fiji, 2018–2019

**DOI:** 10.1371/journal.pgph.0003706

**Published:** 2024-10-10

**Authors:** Edifofon Akpan, Li Jun Thean, Rabindra Baskota, Jyotishna Mani, Maria Mow, Mike Kama, Meciusela Tuicakau, Joseph Kado, Lucia Romani, John Kaldor, Daniel Engelman, Andrew C. Steer, Natalie Carvalho

**Affiliations:** 1 School of Population and Global Health, University of Melbourne, Melbourne, Australia; 2 Murdoch Children’s Research Institute, Tropical Diseases Group, Melbourne, Australia; 3 Department of Pediatrics, University of Melbourne, Melbourne, Australia; 4 Ministry of Health and Medical Services, Suva, Fiji; 5 Telethon Kids Institute, Wesfarmers Centre for Vaccines and Infectious Diseases, Nedlands, Australia; 6 Medical School, University of Western Australia, Nedlands, Australia; 7 Kirby Institute, University of New South Wales, Sydney, Australia; 8 Melbourne Children’s Global Health, The Royal Children’s Hospital, Melbourne, Australia; University of Colorado Anschutz Medical Campus: University of Colorado - Anschutz Medical Campus, UNITED STATES OF AMERICA

## Abstract

Scabies and related bacterial skin and soft tissue infections (SSTIs) are highly prevalent in many tropical, low- and middle-income settings. These skin conditions contribute to higher healthcare costs and burdens on healthcare systems. The Big Skin Health Intervention Fiji Trial (“Big SHIFT”) carried out surveillance for scabies and SSTIs from July 2018 to June 2019 in the Northern Division of Fiji, an area with high prevalence of scabies, prior to a division-wide ivermectin-based mass drug administration (MDA) campaign. Using data from Big SHIFT, we sought to estimate the annual direct healthcare costs of scabies and related SSTIs for the Northern Division and extrapolate these costs to the national level. We categorized SSTIs as being potentially scabies-related or unlikely scabies-related, based on a previous study. The analysis used a health system perspective, with the main resource use categories of outpatient visits, bed days during admissions, medicines, and diagnostic tests. We extrapolated the total annual number of cases and direct healthcare costs for all divisions in Fiji based upon previous scabies and impetigo prevalence data across all divisions. The average cost per PHC presentation for scabies was US$17.7, and for potentially scabies-related SSTI was $18.3. The average cost per hospital admission for a potentially scabies-related SSTI case was $439. The estimated annual healthcare costs of scabies and related SSTIs in Fiji was US$3.0 million, with cost per capita of $3.3. Scabies and related SSTIs lead to a heavy economic burden in Fiji and prevention would reduce these healthcare costs.

## Introduction

Scabies is a contagious and itchy skin infestation caused by the mite *Sarcoptes scabiei* var. *hominis* that can lead to secondary bacterial skin and soft-tissue infections (SSTIs). Scabies promotes bacterial SSTIs by causing breaches in the skin. These SSTIs range in severity from impetigo and other uncomplicated infections which can be generally treated in primary healthcare (PHC) settings [1], to necrotizing fasciitis and other complicated SSTIs usually requiring hospital admission [2]. In 2017, The World Health Organization (WHO) recognized scabies as a neglected tropical disease (NTD) following recommendation from the Strategic and Technical Advisory Group for NTDs [3]. In 2019, the Global Burden of Disease study estimated that the global prevalence of scabies was 2.4%, leading to 4.84 million disability-adjusted life years [4]. The Pacific region has a high scabies burden, comprising eight out of the top ten countries with the highest age-standardized disability-adjusted life-years due to scabies [5].

There is a high burden of scabies and related SSTIs at the population level in Fiji, an island country located in the South Pacific Ocean with a census population of 884,887 people in 2017 [6]. In the Skin Health Intervention Fiji Trial (SHIFT) conducted between 2012–2013, scabies prevalence was measured at 36.4% and impetigo prevalence at 23.4% among residents of three islands of Fiji [7]. In a 2015 nationwide cross-sectional study, scabies was observed in 23.6% of participants surveyed [8]. In Fiji, scabies is often initially treated with traditional medicines, and many individuals only seek medical care for prolonged illness or when a secondary skin infection develops [9]. Thus, the national prevalence of impetigo from the cross-sectional study was high (19.6%). In 2016, the Fiji Government annual Health Status Report reported that SSTIs caused 4.3% of mortality within the country [10].

Mass drug administration (MDA), which involves delivering medications to whole communities, is a promising strategy for the public health control of scabies in endemic areas. In Fiji, two pilot studies were conducted to provide evidence on MDA [7,11]. The Skin Health Intervention Fiji Trial (SHIFT) study demonstrated that ivermectin-based MDA was safe and effective in controlling scabies and impetigo in a study population of approximately 2000 people in Fiji [7]. Subsequently, and as previously reported [11], “Big SHIFT” was designed to determine the effect of ivermectin-based MDA for scabies on serious bacterial complications of scabies, delivered at scale to a whole implementation unit (>100,000 people). The MDA comprised one round of treatment consisting of two doses of ivermectin (7 days apart) delivered to the entire population regardless of the presence or absence of signs and symptoms of scabies. Big SHIFT was delivered to the whole population of the Northern Division of Fiji, one of four primary administrative units of Fiji (2017 census population, 131,914) [6]. The Northern division has the highest prevalence of scabies and impetigo among all Fiji divisions–with 1.3 higher odds of scabies and impetigo compared with those in other divisions [8]. Big SHIFT showed that MDA substantially reduced hospitalizations and primary health care presentations for skin and soft tissue infections.

Healthcare in Fiji comprises a government public healthcare system and a smaller private healthcare sector [12]. Government health facilities provide most healthcare services, which are generally free to the public. Where user fees exist, these are modest and some groups such as children aged less than 15 years are exempted from these charges. Private providers, mainly located in urban areas, charge user fees that are much higher than those in public facilities [12]. Primary health facilities such as nursing stations, health centers, or subdivisional hospitals serve as the entry point to the healthcare system. When required, patients may be referred to a higher-level health facility (divisional and specialized hospitals). In 2019, about US$144 million from the Fiji government’s revenue was allocated to health [13].

There is limited evidence globally regarding the cost of scabies and related SSTIs to healthcare systems. A 2018 cost-of-illness analysis for treatment of crusted scabies among Aboriginal communities in the Northern Territory of Australia found that health care costs per patient diagnosed with crusted scabies were over AU$ 35,000 (or US$ 24,600) [14]. We found no cost-of-illness study on scabies or related SSTIs from the Pacific region or in any low- or middle-income country. Quantifying these costs would contribute to a more accurate estimate of the global burden of scabies, further delineating the benefits of scabies control and informing future evaluation of scabies prevention programs. Therefore, we sought to estimate the annual direct healthcare costs of scabies and related SSTIs in Fiji, a middle-income Pacific country with high burden of scabies.

## Methods

### Study design and setting

This study uses prospectively collected data from the Northern Division of Fiji to estimate the costs of healthcare presentations and hospital admissions among patients with scabies and scabies-related SSTIs prior to the delivery of MDA through Big SHIFT. Costs are calculated using an ingredients-based approach and then extrapolated to the national level using epidemiological data from Fiji. For presentations, the study sites were all primary healthcare settings in the Northern Division. For hospital admissions the study site was Labasa Hospital, a 195-bed hospital located in the divisional capital. This hospital is the referral center for the Northern Division and the only healthcare facility in the division with specialist medical and surgical, and intensive care unit (ICU) services. It is also the only hospital in the division with a microbiology laboratory equipped to process specimens for bacterial culture.

### Study population and data collection

Before MDA, Big SHIFT established a monthly reporting system for presentations of scabies and SSTIs at all public PHC facilities over a 50-week period (from 16 July 2018 to 30 June 2019) [1]. Additionally, data on patients presenting to the outpatient departments at subdivisional hospitals, emergency departments, health centers and nursing stations, and Integrated Management of Childhood Illness (IMCI) clinics were prospectively collected using a dedicated data collection tool. PHC staff also used this tool to collect data on cases diagnosed during school visits and community outreaches. Staff at these PHC settings reported presentations of scabies and potentially scabies-related SSTIs (infected scabies, impetigo, cellulitis, abscess, and severe SSTI). The staff collected data regarding treatment, such as the medication prescribed, surgical procedures performed, referral to a larger health facility, and admission in the health facility.

The trial also carried out prospective surveillance of SSTI admissions at the divisional hospital in Labasa over a 48-week period (between 16 July 2018 and 30 June 2019, with a 2-week break between 24 December 2018 and 6 January 2019) [2]. These scabies and SSTI hospital admissions were prospectively identified by reviewing admission registries and case notes of all newly admitted cases at the hospital, daily. Informed consent was obtained from all patients that were included in the study. The microbiology laboratory records in the hospital were reviewed for skin swabs to identify potential cases for enrolment in our study [15]. Hospital admissions for SSTIs were categorized into two groups: those potentially scabies-related (infected scabies, impetigo, abscess, cellulitis, pyomyositis, necrotizing fasciitis with pure growth of *Staphylococcus aureus* or *Streptococcus pyogenes*, and crusted scabies), and those unlikely scabies-related (wound infections, surgical wound infections, and necrotizing fasciitis without pure growth of *S*. *aureus* or *S*. *pyogenes*) [2].

### Estimation of healthcare costs

#### Costing perspective and categorization

We estimated the mean costs as well as the total annual costs, using extrapolated number of cases in Fiji, to estimate the economic burden at a national level. Costs were calculated from a health system perspective—including the direct costs of outpatient visits to PHC facilities, admissions to PHC facilities, hospital admissions, diagnostic tests, and medicines. We used this perspective because public provision of most outpatient and inpatient care is free in Fiji and user fees are very low compared with the overall health expenditure in government facilities [12]. In line with our costing perspective, we did not consider costs of productivity losses from premature death, SSTI-related work/school absenteeism, or other social impacts.

#### Quantifying resource use

The main resource use categories were health services (outpatient visits and admissions); diagnostic procedures; and medicines (oral, injection, and topical). We measured outpatient visits using the number of presentations in a PHC setting (one outpatient visit per presentation). Our data did not include follow-up visits and repeated presentations for the same symptoms, because the Big SHIFT study did not collect this information. We measured hospital admissions using the number of bed days for admissions to general wards and intensive care unit (ICU). Most PHC presentations were managed on an outpatient basis; however, some cases required admission. We had no data on the length of stay for patients admitted in PHC facilities, so we assumed PHC admissions were for one night only. The number of diagnostic tests was calculated as the sum of skin swabs, blood cultures and tissue cultures taken from patients admitted to the hospital.

To estimate the quantities of medicines, we supplemented the utilization data collected in the trial with relevant antibiotics and treatment guidelines for scabies and SSTIs in Fiji [16,17]. For PHC presentations, we did not have the names of medicines prescribed, but only the dosage form. Therefore, we considered that patients treated with topical medicines were given permethrin cream [16]. We used the average household size in Fiji, 4.2 in 2021 [18], to quantify the number of tubes that would be sufficient to treat the whole family as recommended in the guidelines. For those prescribed an injection, we used benzathine penicillin (for impetigo) or cloxacillin (for cellulitis, abscess, or severe SSTI) as recommended by Fiji antibiotics guidelines [16]. Similarly, those prescribed oral medicines had co-trimoxazole (for impetigo) or flucloxacillin (for the other SSTIs).

The hospital admissions dataset included the name of medication and number of days prescribed during admission and on discharge, but not the dosage. Therefore, we categorized patients into age groups (less than 5 years, 5 to 9 years, 10 to 14 years, and 15 years and above), and then calculated the dosage using the median weight of the average age for each age group based on WHO child growth standards [19]. For instance, if the child was prescribed cloxacillin injection (dosage 50mg/kg up to 2000mg daily every six hours), we assumed the dose was 550mg and the daily dose was 2200mg. Each vial of cloxacillin contains 500mg, so five vials (2500mg) would be sufficient for one day. Finally, where the dosage calculated was greater than the recommended adult dose, the adult dose was used instead. S1 Table details the recommended doses and daily quantities of medicines used in the costing exercise.

#### Estimating mean healthcare costs

We multiplied the resource quantities by their unit costs to derive the cost per case, separately for scabies PHC presentations, potentially scabies-related SSTI PHC presentations, and potentially scabies-related SSTI hospital admissions. PHC presentations include both outpatient visits and admission to PHC facilities. We also calculated mean costs for unlikely scabies-related SSTI hospital admissions. The unit costs of medicines were largely based on procurement prices collected at CWM Pharmacy, and supplemented with other sources, where unavailable (see S1 Table). The unit costs per diagnostic test, outpatient visit, admission to PHC facilities, and hospital admission were obtained from a Fiji costing study [20]. The study estimated the costs of various healthcare services in three facilities—Lautoka (LTK) hospital, Colonial War Memorial (CWM) hospital, and Nausori PHC. We used the average cost per laboratory test as unit cost of diagnostic tests in our study.

The outpatient visits and admission unit cost estimates from the Fiji costing study includes both recurrent costs (staff time and overheads) and capital costs (building, equipment, beds, furniture, and vehicles). We used the cost per outpatient visits in Nausori PHC as unit cost for outpatient visits in our study. The Fiji costing study estimated the costs per bed day for the two hospitals (LTK and CWM), so we used the lower of the values to obtain conservative estimates. Accordingly, we used the cost per bed day in CWM hospital as unit cost for both admission to PHC facilities and hospital ward admissions. Likewise, we used the cost per ICU bed day in LTK hospital as unit cost for hospital ICU admissions. The cost of surgical procedures was not estimated separately because it was captured in the unit cost of admissions in the Fiji costing study [20].

Costs were reported in 2020 United States dollars (US$). Costs obtained in other years and/or different currencies converted to Fijian dollar using the average exchange rate in that year [21], adjusted to 2020 prices using GDP deflator [22], and converted back to US$ using 2020 exchange rates. We assumed scabies to be an acute disease, so we only considered healthcare costs that occurred within one year, and therefore did not apply any discounting for future costs [23].

#### Extrapolating costs to all Fiji divisions

We extrapolated case numbers to all Fiji divisions using the expected annual number of cases in the Northern Division, and the risk ratio (RR) of scabies infestation between the Northern Division and other divisions. The expected annual number of cases in the Northern Division was derived by multiplying the annual risk of cases by the population size.


$$Risk_{Northern}=1-{exp}^{-(Rate\times Time)}$$

$$Cases_{Northern}=Risk_{Northern}\times Population$$

Where *Risk*_*Northern*_ is the risk of cases in the Northern Division, *Rate* is the incidence rate of cases, *Time* is the follow-up time (50 weeks for presentations and 48 weeks for admissions), and *Case*_*Northern*_ is the annual number of cases.

A study by Romani [8] indicated that scabies and impetigo cases were prevalent in the Northern division compared with other Fiji divisions, with an adjusted odds ratio (OR) of 1.3 for each disease. We assumed that the OR for impetigo holds for other potentially scabies-related SSTIs. We converted the OR to risk ratio (RR) using the formula in Zhang and Yu [24], and multiplied this RR WITH the risk in the Northern Division to derive the risk for other Fiji divisions.


$$RR=\frac{OR}{1-Risk_{Northern}+(Risk_{Northern}\times OR)}$$

$${Risk}_{Others}={Risk}_{Northern}\times RR$$

To obtain the expected annual cases in other Fiji divisions, we multiplied the annual risk of cases above with the population size for all other Fiji divisions (752,973 based on 2017 census) [6]. Lastly, we summed up the number of cases for the Northern and the number for other divisions to get the total annual number of cases in all Fiji divisions.


$${Cases}_{Other}={Risk}_{Other}\times Population$$

$${Cases}_{Total}={Cases}_{Northern}+{Cases}_{Other}$$

**Fig 1:**
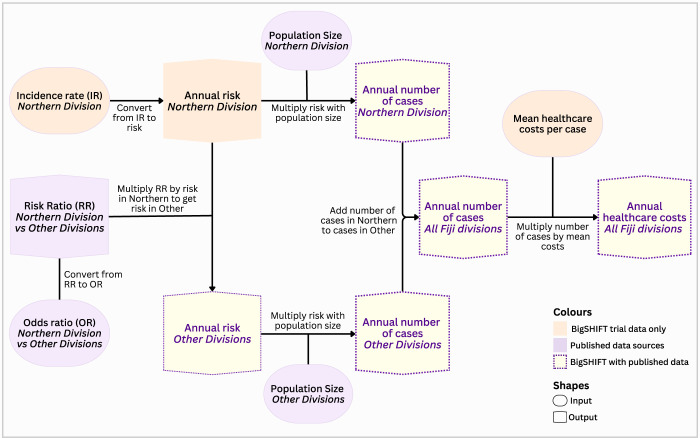
Flowchart for extrapolating cases and costs of scabies and potentially scabies-related SSTI to all Fiji divisions. IR, incidence rate; OR, odds ratio; RR, risk ratio.

These extrapolated number of cases in Fiji were multiplied by the mean costs per case to yield the annual costs. This procedure was carried out separately for scabies PHC cases, potentially scabies-related SSTI cases in PHC, and potentially scabies-related SSTI hospital admissions. Finally, we summed up the annual costs of all PHC and hospital cases to yield the total annual costs for all cases (Fig 1).

### Sensitivity analysis

A one-way sensitivity analysis was conducted to examine how sensitive the cost estimates were to variations in unit costs. The WHO-CHOICE project estimated the cost for inpatient and outpatient health service delivery [25]. WHO-CHOICE unit costs for PHC visits/presentation and ward admission were lower than our base case values, so we used WHO-CHOICE as “low” value and got our “high” values from the costing study (see Table 1). For ICU, used an average of ICU bed day estimates from two hospitals in the Fiji costing study [20] in the base case, so these two estimates served as our low/high value for sensitivity analysis. For laboratory tests, we used wide (±50%) variation to reflect reasonable uncertainty. The results of the sensitivity analysis were reported in a tornado chart. We also did a two-way sensitivity analysis, where we varied two (unit cost) parameters simultaneously; the selected parameters were those that exhibited the highest range of total cost values in the one-way sensitivity analysis.

**Table 1:** Unit costs (in 2020 US$ dollars) used in this study.

Parameter	Base case (low—high)	Source and notes
Cost per diagnostic test	13.0 (11.3–14.8)	Low value from costing study (CWM hospital); high value from costing study (LTK hospital); Base case is average of the two values.
Cost per outpatient visit	11.6 (5.9–29.1)	Base case from costing study (Nausori PHC); low value from WHO-CHOICE (PHC with bed); and high value from costing study (LTK hospital).
Cost per bed day in a hospital ICU	252.4 (119.4–385.5)	Base case from costing study (LTK hospital); Low value uses the cost per bed day in hospital ward; high value from costing study (CWM hospital).
Cost per bed day in hospital ward	56.3 (43.3–61.2)	Base case from costing study (CWM hospital); low value from WHO-CHOICE (tertiary hospital); and high value from costing study (LTK hospital).
Cost per bed day in a PHC ward	56.3 (43.3–61.2)	Same as hospital ward
Cost of oral, injectable, and topical medicines	varies	see S1 Table

CWM, Colonial War Memorial; ICU, intensive care unit; LTK, Lautoka; PHC, primary healthcare; WHO-CHOICE, World Health Organization CHOosing Interventions that are Cost-Effective.

All analyses were conducted using R version 4.3.2 (R Foundation for Statistical Computing, Vienna, Austria).

### Ethics statement

The study was performed as part of the Big SHIFT trial investigating the effects of ivermectin-based MDA for the control of scabies and SSTIs (trial ID: ACTRN12618000461291). Ethical approval for Big SHIFT was granted by the Fiji National Health Research Ethics Review Committee (reference: 2018.38.NOR) and the Royal Children’s Hospital Human Research Ethics Committee in Melbourne, Australia (reference: 38020). The trial obtained written informed consent from all participants or from their parent or legal guardian if they were below 18 years or lack the capacity to provide properly informed consent.

## Results

### Characteristics of presentations and admissions

The expected annual number of PHC presentations in the Northern Division was 3,747 for scabies and 11,292 for potentially scabies-related SSTI presentations (Table 2). Over 80% of PHC presentations were patients of iTaukei ethnicity. The median age was lower for patients presenting to PHCs with scabies, compared with potentially scabies-related SSTIs presentations or admissions. 617 cases of scabies-related SSTI admissions are expected annually in the Northern Division. The average length of stay (bed days) of these admissions was about 7 days. The estimated annual number of unlikely scabies-related SSTI admissions is presented in S2 Table.

**Table 2:** Estimated annual number of PHC presentations and hospital admissions for scabies and scabies-related skin and soft tissue infections in Northern Division, Fiji.

Characteristic	Scabies presentations to PHC^1^	Potentially scabies-related SSTI presentations to PHC^1^	Potentially scabies-related SSTI hospital admissions
Annual total	3747	11292	617
Sex, n. (%)			
Male	1969 (53%)	6179 (55%)	324 (53%)
Female	1755 (47%)	5052 (45%)	293 (47%)
Age, median (IQR)	5 (1, 9)	12 (3, 33)	33 (9, 55)
Age category, n. (%)			
0–4	2066 (55%)	3844 (35%)	119 (19%)
5–14	1263 (34%)	2307 (21%)	74 (12%)
15+	395 (11%)	4995 (45%)	424 (69%)
Ethnicity, n. (%)			
I-Taukei	3168 (86%)	9194 (83%)	316 (67%)
Others	496 (14%)	1916 (17%)	201 (33%)
Residence, n. (%)			
Urban	1772 (47%)	5737 (51%)	NA
Rural	1975 (53%)	5555 (49%)	NA
Bed days, mean (SD)^1^	1.0	1.0	6.9 (6.5)

Notes: Numbers may not add up to totals due to missing values for some characterisitics. SD, standard deviation; NA, not available; PHC, primary healthcare; SSTI, skin and soft tissue infection.

^1^ PHC presentations include both outpatient visits and admissions to PHC facilities. The duration of PHC admissions were assumed to be one night, since there was no available data on length of stay for PHC-admitted patients.

### Healthcare resource use and costs per case

The average cost per presentation from a health system perspective was US$17.7 for scabies and US$18.3 for potentially scabies-related SSTIs (Table 3). Approximately two-thirds of costs of scabies and related SSTI presentations were for outpatient visits. Patients presenting to PHCs with scabies were likely to be prescribed topical medication (81.6% of scabies patients; average costs of $4.0). Those presenting with a potentially scabies-related SSTI were likely to be require oral medicines (83.2% of related SSTI patients; average costs, $2.1). Additionally, those presenting with scabies were less likely than those with potentially scabies-related SSTIs to require admission to PHC facilities (0.1% vs 1.0%) or referral (0.6% vs 1.3%). S3 Table in the supplementary appendix contains the proportion of presentations and admissions requiring each resource use category.

**Table 3:** Mean cost of scabies and potentially scabies-related SSTIs in Northern Division, Fiji.

Category	Scabies presentations to PHC, mean (SD)	Potentially scabies-related SSTI presentations to PHC, mean (SD)	Potentially scabies-related SSTI hospital admissions, mean (SD)
Outpatient visits	11.6 (0.0)	11.6 (0.0)	NA
Ward bed days	<0.1 (1.6)	0.5 (5.5)	378.5 (350.2)
ICU bed days	NA	NA	41.3 (242.0)
Topical medicines	4.0 (1.9)	0.4 (1.3)	NA
Oral medicines	0.7 (1.3)	2.1 (1.9)	1.2 (2.8)
Injection medicines	0.2 (0.6)	1.2 (1.5)	8.3 (10.8)
Diagnostic tests	0.0 (0.0)	0.0 (0.0)	9.9 (7.7)
Mean total costs	17.7 (3.5)	18.3 (6.9)	439.2 (468.3)

Notes: Costs are expressed in in 2020 US dollars. Values in parenthesis are standard deviations. ICU, intensive care unit; NA, not applicable; PHC, primary healthcare; SD, standard deviation; SSTIs, skin and soft tissue infections.

The average cost per hospital admitted case of a potentially scabies-related SSTI from a health system perspective was $439 (see S4 Table for non-scabies-related SSTIs). Approximately 86% of admission costs were for hospital bed days. The average ward bed day cost for potentially scabies-related SSTI admissions was $821. Admission costs are directly related to the mean length of hospital stay, which was 6.9 days for scabies-related SSTIs (see S2 Table for unlikely scabies-related SSTIs). The average ICU bed-day cost was $1,067 among those that required an ICU (3.9% of hospital admissions), or $41 among all admissions. Surgical procedures were required for 63.2% of potentially scabies-related SSTI hospital admissions (commonly incision and drainage, dressing, and debridement) [20]. Injectable medicines were prescribed for a mean duration of about 4.9 days for potentially scabies-related SSTI hospital admissions (oral medicines, about 8.6 days).

### Total annual cases and healthcare costs

Extrapolating the data from the Northern Division to the rest of Fiji, the estimated annual number of PHC presentations for scabies and related SSTIs was 82,183 (Table 4). The estimated number of hospital admissions for potentially scabies-related SSTIs was 3,330 (equivalent to about 23,000 hospital bed days). From a health system perspective, the estimated annual healthcare costs of scabies and related SSTIs in Fiji was US$3.0 million.

**Table 4:** Estimated annual number of cases and costs of scabies and potentially scabies-related SSTIs in Fiji.

Component	Number of cases	Total annual cost ($)	Cost per capita ($)
Scabies presentations to PHC (a)	20,311	359,790	0.41
Potentially scabies-related SSTI presentations to PHC (b)	61,873	1,131,421	1.28
Potentially scabies-related SSTI hospital admissions (c)	3,330	1,462,236	1.65
All scabies and related SSTI presentations (a+b)	82,183	1,491,211	1.69
All scabies and related SSTI cases (a+b+c)	85,513	2,953,447	3.34

Costs are expressed in in 2020 US dollars. PHC, primary healthcare; SSTI, skin and soft tissue infection.

**Fig 2:**
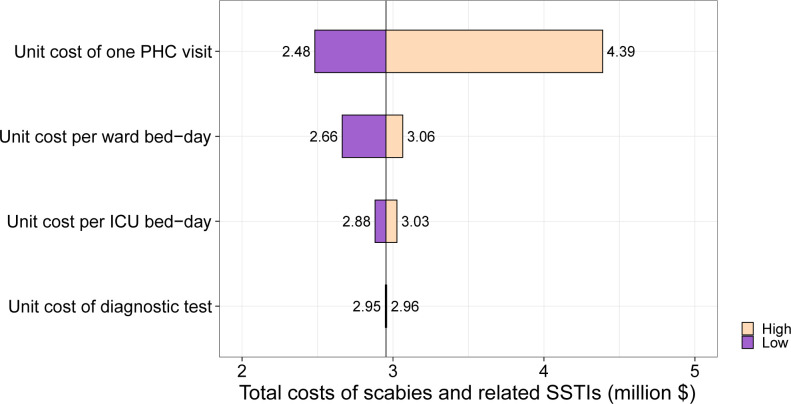
One-way sensitivity analysis on the impact of changing the base case parameter values to low and high values on the total annual healthcare costs of scabies and potentially scabies-related SSTIs in Fiji. Costs are expressed in in 2020 US dollars. Total annual cost is indicated by the black reference line ($2.95 million). See Table 1 for the ranges used for this sensitivity analysis. ICU, intensive care unit; PHC, primary healthcare; SSTI, skin and soft tissue infection.

One-way sensitivity analysis indicated that variations in the cost per outpatient visit had the biggest influence on the total cost of scabies and SSTIs in Fiji (Fig 2). In all sensitivity analyses, the lowest annual cost was at least US$2.5 million. The two parameters that exhibited the highest range of total cost values in the one-way sensitivity analysis were the unit cost of outpatient visit and the unit cost per bed day in a general hospital ward. When these two parameters were varied simultaneously, the annual healthcare costs ranged from US$2.2 to 4.5 million.

## Discussion

Our study provides the first estimate of the healthcare resource use and associated costs of treating scabies and related SSTIs in a highly prevalent middle-income setting prior to an MDA program. We used before-intervention data from the Big SHIFT trial in Fiji and extrapolated these costs to the entire country. The trial provided rich information on PHC presentations (including visits and admissions) and hospital admissions for scabies and related SSTIs in the Northern Division of Fiji. The estimated annual direct healthcare costs of scabies and related SSTIs in Fiji was estimated to be $3.0 million, equivalent to 2.4% of government revenues allocated to health in 2019. Scabies and related SSTIs therefore lead to a heavy economic burden in Fiji, raising the potential benefit of prevention programs such as MDA.

The main resource use category contributing to overall costs was hospital bed days. The estimated 23,000 bed days in our study represents 7.1% of admissions for all divisional hospitals in Fiji in 2017 (3.6% of divisional and specialist hospital admissions). The mean length of stay in our study (6.9 days) was higher than the mean of 4.5 days reported in an Australian pediatric study [26]. It is plausible that delayed detection and normalization of skin infections contribute to complications of scabies. In countries endemic for scabies like Fiji, patients may not seek treatment of scabies unless it creates a significant disturbance to their quality of life [27]. However, the average cost per scabies-related SSTI admission ($439), was much lower than an estimated per-patient cost of US$10,499 for hospital treatment of pediatric scabies and pyoderma in an Australian study in 2019 [28].

The average cost of medicines for treatment of scabies presentations to PHC in our study was $4.0. This amount is more than double the estimated cost of medicines for treatment of outpatient pneumonia in Fiji [29]; in that study, the average cost of medicines ranged from $1.3 for Nausori PHC to $2.6 for CWMH (in 2020 values). One possible explanation for the higher mean cost of medicines in our study is that scabies management involves treatment of household and other close contacts [16,17], which increases the quantity of medications required for a single scabies case. However, previous research indicates that PHC staff do not always treat family members and other close contacts [9]. Therefore, by assuming all household contacts were treated we may have overestimated medication costs. On the other hand, medications prescribed during repeated presentations or follow-up visits were not costed because data on these presentations were not collected in the Big SHIFT study [1]. By not including those presentations in the analysis, we have underestimated medications costs.

Following earlier work [1,2], we categorized specific bacterial SSTI presentations to PHC (infected scabies, impetigo, abscess, and cellulitis) and a range of bacterial SSTI requiring hospital admission (infected scabies, impetigo, abscess, cellulitis, pyomyositis, necrotizing fasciitis) as being potentially related to scabies. It is likely that these assumptions over-estimated the attribution of bacterial skin infection to scabies, and therefore over-inflated the economic burden of scabies-related skin infection. This attribution may be direct (an individual has scabies, and their scabies lesions become infected with bacteria) or indirect (the burden of scabies in the community promotes higher rates of bacterial skin infection within that community and so transmission and exposure to these bacteria is vastly increased). While there are few data to guide the attribution of bacterial SSTIs to scabies in highly endemic settings, two lines of evidence suggest that impetigo is highly associated with scabies in Fiji and other Pacific Island countries. First, the population attributable risk of impetigo to scabies has ranged from 41 to 93% in studies in Fiji and the Solomon Islands [8,30]. Second, substantial reductions in scabies prevalence (~90%) after ivermectin-based MDA have resulted in reductions of 67–75% in impetigo prevalence (without dedicated impetigo treatment) [31,32].

A strength of our study is that we analyzed PHC data obtained from a trial that was conducted among the entire population of the Northern Division of Fiji, including children and the elderly. However, hospital surveillance was only possible at Labasa Hospital, the main referral center for the division. The Northern Division is made up of four subdivisions, and Labasa Hospital is in Macuata subdivision; about 74% of all admissions to Labasa hospital were among residents of Macuata subdivision, and so it is likely that we missed admissions at subdivisional hospitals, thereby underestimating the overall burden. Furthermore, we may have also underestimated the burden by missing SSTI cases that were not recognized by clinicians because clinicians are known to normalize scabies in endemic settings [33]; missing cases that were recognized and treated but not included in patient records; and missing cases in the community because individuals chose traditional medical remedies for scabies treatment [9].

Our analysis used a health system approach, utilizing micro-costing that involved direct quantification and costing of each resource use item. However, we did not consider resource use for managing recurrent SSTI cases, or for containing institutional outbreaks. Furthermore, we did not consider non-healthcare costs such as transportation. Indirect costs were not available to us from the Big SHIFT study, which precluded adopting a societal perspective. It is known that scabies and related SSTIs cause a range of societal impacts, ranging from school and work absence to impacts from stigma [9,27]. Additional limitations relate to our extrapolation approach. We only had data to calculate the odds ratio of impetigo in other divisions of Fiji compared to the Northern Division, so we used impetigo as a proxy for all potentially scabies-related SSTIs. Our approach also assumes that healthcare utilization patterns in other divisions of Fiji were consistent with those observed in the Northern Division.

## Conclusion

Our study contributes to a sparse literature on the direct healthcare costs of scabies and related SSTIs in high prevalence, middle-income settings. We found that scabies imposes a substantial economic loss to the government in relation to costs and healthcare resource utilization. Investment in scabies prevention and control may reduce the direct and indirect cost of scabies treatment in the longer term. Our findings are likely to be relevant to other countries in the Pacific, where the burden of scabies and the costs of treatment may be similar to that of Fiji. Additionally, our estimates provide necessary inputs for modelling the cost effectiveness of public health control interventions for scabies such as MDA.

## Supporting information

S1 Table Unit costs (in US$) and guideline-recommended doses of medicines used in the study. (DOCX)

S2 Table Estimated annual number of unlikely scabies-related skin and soft tissue hospital admissions in Northern Division, Fiji. Notes: Numbers may not add up to totals due to missing values for some characterisitics. SD, standard deviation; NA, not available; SSTI, skin and soft tissue infection.
(DOCX)

S3 Table Healthcare resource utilization for scabies and potentially scabies-related SSTIs in Northern Division, Fiji. ICU, intensive care unit; PHC, primary healthcare; SSTI, skin and soft tissue infection.
(DOCX)

S4 Table Mean costs of unlikely scabies-related SSTIs in Northern Division, Fiji. **Values in parenthesis are standard deviations.** SD, standard deviation; SSTIs, skin and soft tissue infections.
(DOCX)

S1 Data (DTA)

S2 Data (DTA)
