# Supplementary material for: Costs of primary healthcare presentations and hospital admissions for scabies and related skin infections in Fiji, 2018–2019
Source: PLOS Glob Public Health. 2024 Oct 10;4(10):e0003706. doi: 10.1371/journal.pgph.0003706 (PMC11466383; doi:10.1371/journal.pgph.0003706)
Supplement: S1 Table — (DOCX) [file pgph.0003706.s001.docx]

S1 Table. Unit costs (in US$) and guideline-recommended doses of medicines used in the study.

| Medicines | Recommended dosage | Unit cost | Source for costs |
| --- | --- | --- | --- |
| Injection cloxacillin 500mg | 2g (child:50mg/kg up to 2g) 6-hourly | 0.221 | CWM hospital |
| Injection gentamicin 80mg/2mL | 3.2mg/kg up to 320mg daily | 0.034 | CWM hospital |
| Injection penicillin procaine 4 million IU | 1.5 million units (child: 50k/kg) daily | 0.652 | CWM hospital |
| Injection metronidazole 500mg/100mL | 500mg (child: 12.5mg/kg) 12-hourly | 0.894 | CWM hospital |
| Injection erythromycin 1000mg | 500mg (child: 25 mg/kg) 6-hourly | 4.687 | CWM hospital |
| Injection ceftriaxone 1000mg | 2g (child: 50 mg/kg) daily | 0.414 | CWM hospital |
| Injection ciprofloxacin 100mg/50mL | 400mg (child:10mg/kg) 12-hourly | 2.903 | Fiji EML 3^rd^ ed. |
| Injection meropenem 1000mg | 1g (child: 20mg/kg) 8-hourly | 13.36 | CWM hospital |
| Injection penicillin G 4 million IU | 2.4 million units (child: 50k/kg) daily | 0.438 | CWM hospital |
| Oral capsules flucloxacillin 500mg | 500mg (child: 12.5mg/kg) 6-hourly | 0.037 | CWM hospital |
| Oral penicillin 250mg | 500mg (child: 12.5 mg/kg) 6-hourly | 0.013 | CWM hospital |
| Oral capsule amoxycillin 500mg | 1g (child: 25mg/kg) 8-hourly | 0.018 | CWM hospital |
| Oral tablets metronidazole 200mg | 400mg (child: 10mg/kg) 12-hourly | 0.005 | CWM hospital |
| Oral tablets erythromycin 250mg | 500mg (child: 10mg/kg) 6-hourly | 0.028 | CWM hospital |
| Oral capsules doxycycline 100mg | 100mg (child: 2mg/kg) 12-hourly | 0.012 | CWM hospital |
| Oral capsules cephalexin 500mg | 500mg (child: 12.5mg/kg) 6-hourly | 0.034 | UNICEF supply |
| Oral tablets co-trimoxazole 480mg | 960mg (child: 24mg/kg) 12-hourly | 0.007 | CWM hospital |
| Oral susp. flucloxacillin 125mg/5mL (100mL) | 500mg (child: 10mg/kg) 6-hourly | 0.110 | CWM hospital |
| Oral susp. penicillin 125mg/5mL (100mL) | 500mg (child: 12.5 mg/kg) 6-hourly | 1.378 | FCCC authorization |
| Oral susp. amoxycillin 125mg/5mL (100mL) | 1g (child: 25mg/kg) 8-hourly | 0.392 | CWM hospital |
| Oral susp. metronidazole 200mg/5mL (100mL) | 400mg (child: 10mg/kg) 12-hourly | 0.560 | UNICEF supply |
| Oral susp. erythromycin 125mg/5mL (100mL) | 500mg (child: 10mg/kg) 6-hourly | 1.134 | CWM hospital |
| Oral susp. cephalexin 125mg/5mL (100mL) | 500mg (child: 12.5mg/kg) 6-hourly | 0.560 | Same as metronidazole |
| Oral susp. co-trimoxazole 240mg/5mL (100mL) | 960mg (child: 24mg/kg) 12-hourly | 0.290 | CWM hospital |
| Cream permethrin 5% w/w | Topical application for whole family | 1.165 | Mow, et al. |
