# Supplementary material for: Costs of primary healthcare presentations and hospital admissions for scabies and related skin infections in Fiji, 2018–2019
Source: PLOS Glob Public Health. 2024 Oct 10;4(10):e0003706. doi: 10.1371/journal.pgph.0003706 (PMC11466383; doi:10.1371/journal.pgph.0003706)
Supplement: S2 Table — Notes: Numbers may not add up to totals due to missing values for some characterisitics. SD, standard deviation; NA, not available; SSTI, skin and soft tissue infection. (DOCX) [file pgph.0003706.s002.docx]

S2 Table. Estimated annual number of unlikely scabies-related skin and soft tissue hospital admissions in Northern Division, Fiji.

| Characteristic | Unlikely-scabies-related SSTI admissions |
| --- | --- |
| Annual total | 617 |
| Sex, no. (%) |  |
| Male | 114 (59%) |
| Female | 80 (41%) |
| Age, median (IQR) | 35 (21, 54) |
| Age category, no. (%) |  |
| 0-4 | 13 (7%) |
| 5-14 | 23 (12%) |
| 15+ | 158 (82%) |
| Ethnicity, no. (%) |  |
| I-Taukei | 134 (69%) |
| Others | 60 (31%) |
| Residence, no. (%) |  |
| Urban | NA |
| Rural | NA |
| Admitted, no. (%) | 194 (100%) |
| Bed days, mean (SD) | 11.6 (12.5) |

Notes: Numbers may not add up to totals due to missing values for some characterisitics. SD, standard deviation; NA, not available; SSTI, skin and soft tissue infection.
