# Supplementary material for: Costs of primary healthcare presentations and hospital admissions for scabies and related skin infections in Fiji, 2018–2019
Source: PLOS Glob Public Health. 2024 Oct 10;4(10):e0003706. doi: 10.1371/journal.pgph.0003706 (PMC11466383; doi:10.1371/journal.pgph.0003706)
Supplement: S3 Table — ICU, intensive care unit; PHC, primary healthcare; SSTI, skin and soft tissue infection. (DOCX) [file pgph.0003706.s003.docx]

S3 Table. Healthcare resource utilization for scabies and potentially scabies-related SSTIs in Northern Division, Fiji.

| Characteristic | Scabies  presentations to PHC | Potentially scabies-related SSTI  presentations to PHC | Potentially scabies-related SSTI hospital admissions |
| --- | --- | --- | --- |
| Clinic visits | 100.0% | 100.0% | 0.0% |
| Ward bed days | 0.1% | 1.0% | 100.0% |
| ICU bed days | 0.0% | 0.0% | 3.9% |
| Topical medicines | 81.6% | 8.0% | 0.0% |
| Oral medicines | 41.4% | 83.2% | 92.6% |
| Injection medicines | 26.8% | 51.3% | 97.7% |
| Diagnostic tests | 0.0% | 0.0% | 68.0% |

ICU, intensive care unit; PHC, primary healthcare; SSTI, skin and soft tissue infection
