# Supplementary material for: Costs of primary healthcare presentations and hospital admissions for scabies and related skin infections in Fiji, 2018–2019
Source: PLOS Glob Public Health. 2024 Oct 10;4(10):e0003706. doi: 10.1371/journal.pgph.0003706 (PMC11466383; doi:10.1371/journal.pgph.0003706)
Supplement: S4 Table — Values in parenthesis are standard deviations. SD, standard deviation; SSTIs, skin and soft tissue infections. (DOCX) [file pgph.0003706.s004.docx]

S4 Table. Mean costs of unlikely scabies-related SSTIs in Northern Division, Fiji

| Characteristic | Unlikely scabies-related SSTI admissions, mean (SD) |
| --- | --- |
| Clinic visits | 0.0 (0.0) |
| Ward bed days | 632.6 (692.2) |
| ICU bed days | 80.4 (385.4) |
| Topical medicines | 0.0 (0.0) |
| Oral medicines | 1.2 (2.8) |
| Injection medicines | 20.0 (42.6) |
| Diagnostic tests | 11.9 (5.7) |
| Mean total costs | 746.1 (848.3) |

Values in parenthesis are standard deviations. SD, standard deviation, SSTIs, skin and soft tissue infections.
